# Professor Credé’s Clinic in Leipsic

**Published:** 1886-07

**Authors:** W. S. Caldwell

**Affiliations:** Vienna, Austria


PROFESSOR CREDE'S CLINIC IN LIEPSIC.

*To the Editors of the* CHICAGO MEDICAL JOURNAL AND EXAMINER:

GENTLEMEN.—I spent several days at Leipsic on my way from Bremen to Vienna, and was delighted with the cordial manner in which I was received by Professor Credé, though I had nothing but my card as an introduction.

He is sixty-six years old, low in stature, heavy built, wears a thick head of white hair which he combs directly back, moves rapidly, is full of energy, and a thorough believer in the peculiar views that he holds and practices in the lying-in wards of the hospital of which he has charge.

I saw laparatomy performed twice by Dr. Sänger, Professor Credé's assistant, who has lately become quite celebrated throughout Europe by his successes in doing the caesarean section. These cases were for the removal of the ovaries for the relief of uterine fibroids.

The Lister spray was kept playing in the room one hour before the operations began, but was removed as soon as the patient was narcotized and everything ready for the operation.

The incision in the median line was about three inches long, the ovaries were ligatured with silk, the stump was cauterized slightly with the thermo-cautery, dusted over with iodoform, and the incision in the median line closed by stitches put in about one-fourth of an inch apart, the line of union being covered with the same drug followed by a thick layer of iodoform gauze and absorbent cotton. In answer to the inquiry as to the benefit derived from this treatment in this class of cases, I was told that so far it had been good, that while the tumor in some cases did not lessen in size, it ceased to grow and the hæmorrhage being arrested the patients generally rapidly improved.

I was fortunate in visiting Professor Credé just at the time when he was giving his lectures on his management of the afterbirth.

After the clinic I always followed the Doctor around the wards to visit and examine the women lately delivered. Every case was carefully looked over, and the card that recorded the temperature and pulse was closely inspected. Each patient had on a large cloth pinned in the manner that a diaper is applied to an infant. Beneath this cloth, covering the entire genitals, was a thick layer of absorbent cotton.

He unpinned the diaper, took off the cotton, smelled of it, asked how long it had been on, and directed as to how frequently it should be applied in the future. Besides this he examined the contour of the womb through the abdominal walls, usually made friction over it for a few moments with his hand, and if the organ appeared larger than he thought it ought to be, gave the patient directions how to repeat at stated intervals the same process herself. Among the cases that we passed there was one in which the cotton removed smelled terribly. I asked, "Professor, is this the way you manage all your cases, including the one with foul discharge?" His reply was that this was his entire treatment.

Fearing still that he did not fully comprehend my meaning, I asked again, "What do you do to counteract this bad odor." His reply was, "*I just let it stink.*"

I asked him farther, how often he found his pressing out process for the removal of the afterbirth to fail him, making it necessary to introduce the hand into the womb or vagina to accomplish the same. Turning to his assistant, who is full of figures and statistics—and telling him to answer me that question, the reply was only three times in the last year in a series of nearly 600 deliveries. He farther explained, that in none of these three cases was the labour a normal one. One was a case of placenta prævia, one a case of miscarriage at six months, and the other followed the removal of a dead fœtus with extreme uterine inertia. Professor Credé has just had issued from the press a work entitled, "Gesunde und Kranke Wöchnerinnen."

After seeing the man himself and something of his practice I have read with more than ordinary interest this work, which he tells us contains the carefully kept records as to pulse, temperature, etc., of over 7,000 patients, running through a period of over twenty years. Among the fundamental facts with which the Doctor begins this work, is that an increase of temperature and pulse-rate is not always a sign of the existence of a true pathological process requiring our active interference, disturbances of the nervous system, derangements of the alimentary tract, slight inflammatory processes in the mammary glands, are among the causes that may increase the bodily temperature and the pulse frequently, without being a source of anxiety to the attending physician.

Again Professor Credé says that a solution of continuity of the surface of the genital tract is an almost universal outcome of every case of childbirth.

To avoid as much as possible the wounding of this canal, and especially the mouth of the womb, he says that all *internal* examinations should be forbidden or only resorted to in extreme cases. Lying-in women should never be used as material for the instruction of students, where such instruction includes a vaginal examination. To be able to make your diagnosis as to the presentation and position of the child, by external manipulation alone, is the great landmark that separates the obstetrics of the past from that of the present.

During the last year he has found it necessary to examine the womb seven times only *per vaginam*, to assist diagnosis, and often two to four weeks will elapse without a single vaginal examination being made in the wards of his hospital. In fact, he proposes to limit such examinations to cases that require manual or instrumental assistance. Having avoided as much as possible the wounding of the genital tract by abstaining from manual examination we still have a more or less wounded surface as a natural result of the pressure that has been brought to bear upon the parts during the passage of the child through the womb and vagina.

"How are you going to manage this wound that is hidden from view and that cannot be treated upon the general principles that underlie the management of wounds in surgical practice?" His answer is, "*Let it alone.*" To vindicate this practice he does not believe in the autogenic origin of effete matters inside the body that are capable of producing the contamination of the blood by being absorbed by these open wounds that follow labour.

In other words the lochial discharge, though varying vastly in quality and quantity, only becomes a source of septic poisoning to the patient when a materies morbi is allowed to gain access to the genital tract through the medium of the hand of the accoucheur or some form of instrument introduced into the same.

In accordance with this view he takes no pains to separate patients who are suffering from puerperal processes from those lately confined but who are perfectly well. As long as the septic process is local in character your prognosis is favorable, but as soon as you have "allgemeine vergiftungen" your case is one that will result almost assuredly fatally.

Where blood-clots and portions of the membranes fail to come away with the afterbirth he thinks it best to let them alone and allow them to pass spontaneously, rather than remove them either manually or instrumentally, thus saving the patient the great danger of being poisoned by the access of germs to the vaginal or uterine tract.

He gives several cases to illustrate the bad consequences of introducing the hand or fingers into the vagina, among them one of a woman who had a premature labour, the child only living 24 hours. She did well until the 9th day, at which date one of the assistants (unauthorized) introduced his hand into the vagina and finding the womb open and a clot of considerable size inside of it, he proceeded to remove the same.

Within a few hours after this seemingly simple operation the patient was taken with a severe chill, followed by a high fever and died on the 17th day after her confinement. The post mortem showed the entire inner surface of the womb to be covered by a pyogenic membrane, the uterine veins engorged and containing pus and a lobular pneumonia as a result of a secondary metastatic process.

The only treatment that this patient received was quinia, wine and camphor. In a rather extensive acquaintance with obstetricians and gynæcologists of Europe one finds unfortunately the most of them astride of a hobby which they ride through good and evil report. Admitting Professor Credés idea, that we generally interfere too much in the management of our cases of labour, yet in the case above described would not a deviation from the plan in the way of washing out the woman's womb as soon as the first signs of fever, etc., made their appearance have added greatly to her chances of recovery? His antisepsis consists in keeping the external parts clean, paying no attention to anything within the vaginal orifice. To facilitate this he cuts the hair around the genitals of the woman short with the scissors at the time of or just before her confinement.

Speaking of the use of quinia and digitalis in the treatment of the puerperal process he says that they are totally worthless, or to quote, "dass sie auf den Vergiftungsvorgang nicht den geringsten Einfluss ausüben."

Ether and camphor hypodermically and wine and cognac by the mouth are the remedies upon which he relies. Though perhaps I have already extended this article to a length that will encroach too much on the space that I ought to occupy in the columns of your JOURNAL, I trust that the following addition from the clinic of Carl Braun bearing on the subjects that I have discussed will be thought of sufficient interest to warrant their insertion.

A week ago a woman was brought into Carl Braun's clinic and placed upon the table with the following history. Twenty-four hours before this time she had been delivered, the labour natural and the after-birth following soon after the birth of the child, but the membranes did not pass away.

She had just had a severe chill and her temperature was 40.6° R.

A large blunt curette was introduced into the womb, the retained membranes were scraped away and the entire inner surface of the womb was *gently* scraped out.

The womb was now washed out with two litres of a solution of bichloride of mercury, one part to 4,000, and this was followed immediately by a second washing out with thymol, one part to 1,000.

The last washing was done to prevent the possibility of mercurial poisoning that might have followed the use of the first remedy. Eighteen hours after this treatment this patient's temperature fell to normal, and one week after this operation she walked into the clinic to show us that she was entirely well. In connection with this case Braun remarked that they had lost a case this spring whose initial symptoms and history had been similar to those of this patient, but in which the active treatment pursued in this last case was not instituted until three days after her chill, and that during this time the septic poisoning had become generally disseminated through her system and no local treatment would arrest the process. Professor Braun, while lecturing on these cases, took occasion to refer to the Credé method of the management of the after-birth, and held that in practicing it you would frequently fail to get the *entire* secundines, that, although the after-birth might come away, the membranes might still remain in the uterus.

Farther than this, where portions of the membranes remained in the womb, his plan was to remove them at once, and if it could not be done without the blunt curette such removal should always be followed by a thorough washing out of the womb with an antiseptic solution. I have some interesting surgical notes from the clinic of Professor Tiersch, of Leipsic, which I shall include in my next letter.

W. S. CALDWELL.

VIENNA, AUSTRIA,

May 29, 1886.

